# Human adenoviruses associated with respiratory illness in neonates, infants, and children in the Sousse area of Tunisia

**DOI:** 10.1002/jmv.26375

**Published:** 2020-08-13

**Authors:** Ines Brini, Aida Guerrero, Issaad‐Kawther Ezzine, Dorothea Orth‐Höller, Benjamin Hetzer, Reinhard Würzner, Olfa Hazgui, Imene Handous, Sonia Nouri‐Merchaoui, Jihene Bouguila, Nabiha Mahdhaoui, Lamia Boughamoura, Zahra Malekshahi, Dorothee von‐Laer, Naila Hannachi, Jalel Boukadida, Heribert Stoiber

**Affiliations:** ^1^ Faculty of Pharmacy of Monastir University of Monastir Monastir Tunisia; ^2^ Laboratoire de Microbiologie, Unité de Recherche Caractérisation Génomique des Agents Infectieux UR12SP34, Hôpital Universitaire Farhat Hached Sousse Université de Sousse Sousse Tunisie; ^3^ Faculté de Médecine de Sousse Université de Sousse Sousse Tunisie; ^4^ Medical University of Innsbruck Innsbruck Austria; ^5^ Institute of Virology Medical University of Innsbruck Innsbruck Austria; ^6^ Laboratoire de Génétique, Biodiversité et Valorisation des Bio‐ressources, Institut Supérieur de Biotechnologie de Monastir Université de Monastir Monastir Tunisie; ^7^ Institute of Hygiene and Medical Microbiology Medical University of Innsbruck Innsbruck Austria; ^8^ Department of Pediatrics Medical University of Innsbruck Innsbruck Austria; ^9^ Service de Néonatologie, Hôpital Universitaire Farhat Hached Sousse Université de Sousse Sousse Tunisie; ^10^ Service de Pédiatrie, Hôpital Universitaire Farhat Hached Sousse Université de Sousse Sousse Tunisie

**Keywords:** acute respiratory infections, coinfections, genotyping, human *Adenovirus*, severity, Tunisia

## Abstract

**Background:**

The human *Adenovirus* (HAdV) is a common agent of acute respiratory infections (ARIs). Its clinical impact in immunocompetent children and in the context of coinfections remains unclear in Tunisia.

**Material and methods:**

HAdV‐ARIs were studied in hospitalized patients from birth to the age of 5 years from 2013 to 2014. Clinical and demographic characteristics, coinfections, and molecular characterization of HAdV were established.

**Results:**

HAdV‐positivity was detected in 114/583 specimens (19.6%) including 6.1% single infections and 93.9% coinfections. Adenoviral coinfections mostly comprised human *Rhinovirus* (50.9%), *Streptococcus pneumoniae* (34.2%), human *Respiratory Syncytial virus A/B* (29.8%), and human *Coronaviruses* (21.9%). HAdV infection was predominant in the pediatric population (25.0% vs 10.0% in neonates, *P* < .001) and peaked in February 2014 (21.1%). HAdV severity of pediatric cases is characterized by low saturation of oxygen (<94%, 33.8%, *P* = .05) and long duration of oxygen support (≥5 days, 32.7%, *P* = .02). Severe HAdV infections were described with S. *pneumoniae* coinfection, which seemed to increase the risk of death. HAdV genotyping identified HAdV‐C as the most common species. Severe ARIs were observed in all HAdV‐identified types. Phylogenetic analysis revealed that sequences were variable suggesting the circulation of different HAdV strains sharing more similarities to strains circulating in Europe or Asia than those from Africa.

**Conclusion:**

This first molecular study of HAdV in Tunisia demonstrated that it has an important role in severe ARIs with HAdV‐C being the most common species. *S. pneumoniae* codetection seems to increase the severity of HAdV‐ARIs.

## 1 INTRODUCTION

Acute respiratory infections (ARIs) are a significant cause of childhood mortality and morbidity worldwide.^1^ Various respiratory pathogens are identified as causative agents for respiratory symptoms including the human *Adenovirus* (HAdV). This pathogen is classified among the Adenoviridae family and belongs to the genus *Mastadenovirus*. It is a ubiquitous nonenveloped virus of medium‐sized double‐stranded‐DNA ranging from 34 kb to more than 37 kb, which encodes around 40 genes.^2^

HAdV has been divided into seven species (A‐G) based on a biological criteria and DNA homology. Each species comprises multiple genotypes. According to the Human *Adenovirus* Working Group, the collaboration between adenoviral researchers and the National Center for Biotechnology Information (NIH)/GenBank, up to 90 genotypes of HAdV have been described. These genotypes were designated with a number according to the chronological order of identification.^3^ The distribution of HAdV genotypes is variable, depending on geographical, environmental, and meteorological characteristics. Some strains may have a higher epidemic potential.^4^ Several species were found to be associated with different clinical profiles. Those most common genotypes implicated in ARIs belong to HAdV‐C (types 1, 2, 5, and 6), HAdV‐B (types 3 and 7), and HAdV‐E (a single type 4) species. Severe and fatal adenoviral diseases were found to be caused by HAdV‐B types 14, 21, and 55.^5^ However, HAdV circulating genotypes in the context of ARIs have not yet been described in Tunisia.

HAdV was found to be one of the causative agents of tonsillopharyngitis, conjunctivitis, pneumonia, gastroenteritis, hepatitis, and hemorrhagic cystitis. In terms of ARIs, this virus causes between 5% and 15% of the overall respiratory illnesses in children aged below 4 years.^6^ Severe or life‐threatening respiratory diseases in immunocompromised cases were frequently reported in the context of adenoviral infection. In addition, HAdV severe infection was also described in immunocompetent patients.^7^ Nonetheless, its detection in respiratory specimens does not necessarily identify HAdV as the causative agent of ARIs but may be related to reactivation or latency, especially, when highly sensitive molecular detection tests are used.^8^ Conversely, some authors have found that there is enhanced severity and worse outcomes in children with mixed respiratory viral infection as compared with those with single infections.^9^ Thus, the role of adenoviral coinfections in ARIs remains controversial and warrants additional research.

The detection of HAdV and the identification of its pathogenicity in ARIs were poorly reported in North Africa.^10^, ^11^, ^12^, ^13^ Although in Tunisia HAdV‐ARIs were previously described,^14^, ^15^, ^16^, ^17^ the molecular characterization of this pathogen was only performed in non‐ARIs.^18^, ^19^, ^20^, ^21^ This study aimed to assess the severity of HAdV and detected coinfections in neonates, infants, and children hospitalized for ARIs in a Central‐East region of Tunisia. The molecular characterization of HAdV was determined. This study highlights the importance of HAdV detection and its coinfections in ARIs and provides the first starting point on the circulation of different genotypes in Tunisia. Such evidence will promote the initiation of preventive measurements against the circulation of HAdV and the most prevalent coinfected pathogens in the community.

## 2 MATERIALS AND METHODS

### 2.1 Ethics and study population

This study did not involve any human experimentation. The study protocol was approved with formal authorization from the Scientific and Ethical Committee of the “Hôpital Universitaire Farhat Hached (CHU‐FH) Sousse, Tunisie,” approval number‐Institutional Review Board‐00008931, and provided by the Office for Human Research Protections. This cross‐sectional study covering the period of 1 October 2013 to 31 December 2014 included patients from birth to 5 years of age who were hospitalized for ARIs in the neonatology and pediatric wards of the “CHU‐FH Sousse, Tunisie.” The diagnosis of ARIs was carried out according to the recommendations of the Tunisian Society of Pediatrics in 2013.^22^ Patients’ data including demographic characteristics, medical history, clinical manifestations, and bacteriology tests, were extracted from medical charts.

### 2.2 Detection of HAdV and other common respiratory pathogens

Nasopharyngeal aspirates were obtained within 24 hours after admission and transported to the Laboratory of Microbiology at the same hospital. Samples were diluted in phosphate‐buffered saline and centrifuged for 10 minutes/3584 ×* g*/+4°C. Using appropriate transport conditions, supernatants were transferred to the Innsbruck Medical University, Austria, where they were directed for the pathogenic genomic detection, bacterial identification, and typing and sequencing reactions.

#### 2.2.1 Multiplex detection of respiratory pathogens by real‐time reverse‐transcription polymerase chain reaction

A magnetic‐particle technology for automated isolation of total nucleic acids was executed using the QIAsymphony Sp automate (QIAGEN. Cat No. /ID: 9001297; Hilden; Germany) and the QIAsymphony DSP virus/Pathogen Mini Kit (QIAGEN; Vienna; Austria). A final volume of 150 µL of extracted nucleic acids was obtained and used for the genomic detection of respiratory pathogens by a multiplex quantitative real‐time reverse‐transcription polymerase chain reaction (qRT‐PCR) with the Fast Track Diagnostics (FTD) Respiratory pathogens 21 Kit (FTD‐2‐64; Luxemburg S.à.r.l.; 29; rue Henri Koch; L‐4354 Esch‐sur‐Alzette). This is a qualitative in vitro diagnostics system generating five‐tube multiplex for the detection of: *Influenza A virus* (InfV‐A); *Influenza B virus* (InfV‐B); *Influenza A(H1N1) virus* swine‐lineage (InfV‐A(H1N1)swl); Human *Rhinovirus* (HRV) in the first mixture, Human *Coronavirus* NL63 (HCoV‐NL63); Human *Coronavirus* 229E (HCoV‐229E); Human *Coronavirus* OC43 (HCoV‐OC43); Human *Coronavirus* HKU1 (HCoV‐HKU1) in the second mixture, Human *Parainfluenza virus 2* (HPIV‐2); Human *Parainfluenza virus 3* (HPIV‐3); Human *Parainfluenza virus 4* (HPIV‐4); internal control in the third mixture, Human *Parainfluenza virus 1* (HPIV‐1); Human *Metapneumovirus A/B* (HMPV A/B); Human *Bocavirus* (HBoV); *Mycoplasma pneumoniae* in the fourth mixture, and Human *Respiratory Syncytial virus A/B* (HRSV A/B); HAdV; Enterovirus genus (EV); Human Parechovirus genus (HPeV) in the fifth mixture, using specific primers and probes. The presence of pathogen sequences is detected by an increase in fluorescence (relevant dual‐labeled probe) which is interpreted by the cycle threshold (Ct) value.

#### 2.2.2 S*treptococcus pneumoniae* genomic detection

*S. pneumoniae* genome detection was performed by the 16S ribosomal DNA qPCR and using the Light Cycler Fast Start DNA Mas (#03003248001) kit as per manufacturer's instructions. The primers and probe were designed by Corless and collaborators.^23^

For all reactions of HAdV detection and other respiratory agents, PCR mixtures and programs were previously provided.^15^, ^16^

### 2.3 HAdV typing, sequencing, and phylogenetic analysis of the partial hexon gene

#### 2.3.1 Genotyping of adenoviruses

Table 1 shows the primer sets used for HAdV molecular identification. Typing reactions were assessed starting from a first PCR (1st PCR) using universal primers designated according to the hypervariable region (HVR) of the hexon gene for all HAdV prototypes (primers set 1). A nested PCR (1st nested PCR) for the typing of HAdV‐C2; ‐B3; ‐E4; ‐B7; and ‐B11 was carried out (primers sets 2‐6). For HAdV‐B14 identification, a second PCR (2nd PCR) was performed using primers sets 8 and 9, respectively. A third PCR (3rd PCR) was done for the detection of HAdV‐B55 (primers set 10).

**Table 1:** Complete primer sets targeting the fraction/total hexon‐coding region of human *Adenovirus* and used for the typing of the most common Human *Adenovirus* prototypes

Set/Ref	Primer	HAdV	Target	Region	PCR product (bp)	Sequence (5′‐3′)
***1*** **(37)**	HVR‐F	Conserved	18473‐18495	HVR of hexon gene (1st PCR)	1684	CAGGATGCTTCGGAGTACCTGAG
	HVR‐R		20157‐20132			TTTCTGAAGTTCCACTCGTAGGTGTA
***2*** **(38,39)**	AdV2‐F	HAdV‐C type 2	18858‐18883	Partial hexon gene (1st nested PCR)	300	GCCGCAGTGGTCTTACATGCACATC
	AdV2‐R		19158‐19136			CAGCACGCCGCGGATGTCAAAGT
***3*** **(37)**	Ad3‐F	HAdV‐B type 3	18933‐18961		313	AAGACATTACCACTACTGAAGGAGAAGAA
	Ad3‐R		19246‐19224			CGCTAAAGCTCCTGCAACAGCAT
***4*** **(37)**	Ad4‐F	HAdV‐E type 4	18665‐18686		323	AGCAAAATGCATACCTTTGGGG
	Ad4‐R		18988‐18967			ATAGTTAGGAGTGGTGGCGGCG
***5*** **(37,40)**	Ad7‐F	HAdV‐B type 7	18890‐18911		300	GGGAAAGACATTACTGCAGACA
	Ad7‐R		19190‐19171			AAAAAGCGTCAGCAGCTTCT
***6*** a **(41)**	AdV11‐F	HAdV‐B type 11	19171‐19187		436	CAAGTTCCGAAGCTAAT
	AdV11‐R		19607‐19591			ACCCTGTCCGATCTCAC
***7*** **(42–48,40)**	AdnU‐S’‐F	Conserved	20743‐20762	Partial hexon gene	955	TTCCCCATGGCNCACAACAC
	AdnU‐A‐R		21698‐21679			GCCTCGATGACGCCGCGGTG
***8***	HVR‐F		18473‐18495	Hexon gene (2nd PCR)	3225	CAGGATGCTTCGGAGTACCTGAG
	AdnU‐A‐R		21698‐21679			GCCTCGATGACGCCGCGGTG
***9*** b **(48,49)**	AdV14‐5F	HAdV‐B type 14	19670‐19689	Partial hexon gene (2nd nested PCR)	327	CGTCCAATGTCACTCTTCCA
	AdV14‐6R		19997‐19978			CCGAGGGAACTCTGTAGCAC
***10*** c **(51)**	AdV55‐2F	HAdV‐B type 55	19672‐19691	Hexon gene (3rd PCR)	1541	ATACACCCCGTCCAATGTCA
	AdV55‐2R		21213‐21194			CGCTTATCGTAGGTTCCCAA

Abbreviations: HAdV, human *Adenovirus*; HVR, hypervariable region; PCR, polymerase chain reaction.

a The nucleotide positions were designated according to the prototype strain of AdV‐11 Slobitski strain (GenBank accession no. AF532578).

b Primers were designated according to the prototype strain of HAdV‐14 de Wit (GenBank accession no. AY803294).

c The nucleotide positions indicated are thoseof the HAdV‐55 strain (GenBank accession no. FJ643676).

#### 2.3.2 Sequencing of HAdV positive specimens

PCR for sequencing a partial region of the hexon gene specific to all HAdVs prototypes and containing the HVR‐7, was performed according to Sarantis et al.^24^ In addition to Tunisian samples, two specimens from Innsbruck were used as control for the optimization of HAdV sequencing reaction (see Table, Supplemental Digital Content 1 that summarizes the HAdV reference sequences targeting a partial hexon gene and control specimens used to generate the phylogenetic tree). Each specimen was analyzed based on Sanger sequencing technology using AD1 and AD2 primers,^24^ accordingly.

#### 2.3.3 Analysis of nucleotide sequences and construction of the phylogenetic tree

Forward and reverse sequences were assembled, examined for ambiguities, and aligned using default settings of “Clustal W” implemented in BioEdit v.7.2.5.^25^ Sequences were trimmed to generate a standard 638 nucleotide‐long fragment then compared with HAdV Hexon gene sequences available on Genbank to estimate their identities. The comparison was performed using the BLASTn program from the US National Center for Biotechnology Information. Sequences of relevant prototypes relative to circulating strains worldwide and which have significant homology percentages with our sequences (≥98%) were downloaded and used as reference sequences (Supplemental Digital Content 1).

For Bayesian Inference, Bayes v.3.2.2^26^ was used under a mixed evolution model. Four independent runs were conducted for 10^6^ generations, sampling every 1000. The first 25% trees were discarded as default burn‐in and a majority rule consensus tree was calculated from the remaining trees. The obtained topology and the posterior probabilities of each node were displayed on FigTree v.1.4.0.^27^

### 2.4 Data analysis and statistics

The Ct‐value corresponds to the number of cycles required for the fluorescent signal, determined by the accumulation of a fluorescent signal during qPCR/qRT‐PCR to cross the threshold and indicating viral loads in specimens. Data were analyzed for statistical significance with the Statistical Package for the Social Sciences (IBM SPSS Statistics, v.24.0) using Pearson's *χ* ^2^ test or Fisher's exact test (where cell counts <5 were encountered). The odds ratio (OR) and 95% confidence interval (95% CI) were calculated. The binary logistic regression model was applied when the test exceeded two tails. A value of *P* ≤ .05 is considered statistically significant. Continuous variables were represented using the mean ± SD or median interquartile range. The categorical variables were described using percentages. A subgroup analysis was carried out to determine *P*‐values between the groups by pairwise comparison of the subgroups. The statistical calculations between HAdV coinfections and clinical data/severity of respiratory infection were uniquely considered for an effect size equal to or above 25 based on the minimum relevant effect size.^28^

## 3 RESULTS

### 3.1 Demographic data of patients admitted for ARIs between 2013 and 2014

A total of 583 subjects from the neonatology ward (0‐45 days, 36.2%) and the pediatric ward (45 days‐5 years, 63.8%), of which 59.8% were infants (45 days‐2 years) and 40% were children aged 2 to 5 years, hospitalized for ARIs (male predominance, sex‐ratio = 1.7) were enrolled. The majority of hospitalizations for ARIs occurred in winter months (46.8% between January and March 2014). All patients had lower ARIs (LARIs) and 18.4% of cases suffered, in addition, from upper ARIs (UARIs). Table 2 summarizes patients' medical data. Some severity factors such as long duration of hospitalization of more than 7 days (75.8% in neonates and 45.2% in the pediatric population) and the need for oxygen support (87.4% in neonates and 31.4% in the pediatric population), were identified. During hospitalization, 6% of cases were fatal, comprising predominately infants and children.

**Table 2:** Characteristics of Human *Adenovirus* infection detected in the neonatology and pediatric environments in Farhat Hached University‐Hospital of Sousse, Tunisia between October 2013 and December 2014

Data specific to the neonatology population					Total	HAdV−	HAdV+	*P*‐value,^a^ OR (95% CI)^b^	
Total, no. (%)					**211 (36.2)**	**190 (90.0)**	**21 (10.0)**	**…**	
Demography and medical history	Age groups, d		*≤10* *		36 (17.0)	29 (80.5)	7 (19.5)	**.04**	**1**
			*10‐45*		175 (83.0)	161 (92.0)	14 (8.0)		**0.36 (0.13‐0.97)**
	Feeding		*Breast* *		39 (32.8)	36 (92.3)	3 (7.7)	.53	
			*Artificial*		33 (27.7)	30 (90.9)	3 (9.1)		
			*Mixed*		47 (39.5)	40 (85.1)	7 (14.9)		
	Prematurity^c^		*No (37‐42)* *		62 (55.9)	55 (88.7)	7 (11.3)	.87	
			*Yes (≤37)*		49 (44.1)	43 (87.8)	6 (12.2)		
	NRDS (*no* *, *yes*)				21 (17.8)	20 (95.2)	1 (4.8)	.45	
Side symptoms/other biological tests	Body T° (°C)			*<36* *	5 (4.3)	5 (100.0)	0	.89	
				*36‐37.5*	64 (54.7)	55 (86.0)	9 (14.0)		
				*37.6‐38*	24 (20.5)	22 (91.7)	2 (8.3)		
				*38.1‐39*	23 (19.7)	21 (91.3)	2 (8.7)		
				*>39*	1 (0.9)	1 (100.0)	0		
	Digestive signs (*no* *, *yes*)				27 (22.7)	21 (77.3)	6 (22.2)	**.02**	**1**
									**4 (1.1‐19.3)**
	CRP (mg/L)			*<6* *	87 (73.7)	76 (87.4)	11 (12.6)	.94	
				*6‐20*	8 (6.8)	7 (87.5)	1 (12.5)		
				*21‐50*	16 (13.6)	15 (93.75)	1 (6.25)		
				*>50*	7 (5.9)	7 (100.0)	0		
Predictive severity symptoms	Duration of hospitalization, d			*1‐7* *	28 (24.1)	25 (89.3)	3 (10.7)	>.99	
				*8‐15*	33 (28.4)	29 (87.9)	4 (12.1)		
				*>15*	55 (47.4)	49 (80.1)	6 (10.9)		
	Ventilation (*no* *, *yes*)				15 (12.6)	14 (93.3)	1 (6.7)	>.99	
	Duration of ventilation, d			*≤3* *	4 (28.6)	4 (100.0)	0	>.99	
				*>3*	10 (71.4)	9 (90.0)	1 (10.0)		
	Oxygen support (*no* *, *yes*)				104 (87.4)	92 (88.5)	12 (11.5)	>.99	
	Death (*no* *, *yes*)				12 (10.1)	11 (91.7)	1 (8.3)	>.99	
Data specific to the pediatric population								
Total, no. (%)					**372 (63.8)**	**279 (75.0)**	**93 (25.0)**	**…**	
Demography and medical history	Age groups	*45 d‐3 mo* *			159 (42.7)	131 (82.4)	28 (17.6)	**.01**	**1**
		*4‐6 mo*			67 (18.0)	45 (67.2)	22 (32.8)		**0.37 (0.19‐0.73)**
		*7‐12 mo*			88 (23.7)	66 (75.0)	22 (25.0)		**0.86 (0.41‐1.8)**
		*13‐60 mo*			58 (15.6)	37 (63.8)	21 (36.2)		**0.58 (0.28‐1.2)**
	Asthma (*no* *, *yes*)				35 (9.4)	26 (74.3)	9 (25.7)	>0.99	
	Feeding			*Breast* *	52 (29.9)	42 (80.8)	10 (19.2)	.23	
				*Artificial*	21 (12.1)	17 (81.0)	4 (19.0)		
				*Mixed*	101 (58.0)	70 (69.3)	31 (30.7)		
	Prematurity (*no* *, *yes*)				72 (19.4)	53 (73.6)	19 (26.4)	.76	
Side symptoms/other biological tests	Anemia (*no* *, *yes*)				192 (54.2)	143 (74.5)	49 (25.5)	.55	
	Gastroenteritis (*no* *, *yes*)				6 (1.6)	1 (16.7)	5 (83.3)	**<.01**	**1**
									**15.8 (1.8‐137)**
	CRP (mg/L)			*<20* *	229 (65.6)	176 (76.9)	53 (23.1)	.46	
				*≥20*	120 (34.4)	88 (73.3)	32 (26.7)		
Associated ARIs	Rhinitis (*no* *, *yes*)				79 (21.2)	59 (74.7)	20 (25.3)	.96	
	Laryngitis (*no* *, *yes*)				18 (4.8)	12 (66.7)	6 (33.3)	.40	
	Pharyngitis (*no* *, *yes*)				10 (2.7)	7 (70.0)	3 (30.0)	.71	
Predictive severity symptoms	Saturation of O_2_ < 94% (*no* *, *yes*)				71 (19.3)	47 (66.2)	24 (33.8)	**.05**	**1**
									**1.7 (0.9‐3.0)**
	Duration of hospitalization, (days)			*0‐3* *	56 (15.1)	44 (78.6)	12 (21.4)	.58	
				*4‐7*	148 (39.8)	107 (72.3)	41 (27.7)		
				*>7*	168 (45.2)	128 (76.2)	40 (23.8)		
	Admission to ICU (*no* *, *yes*)				47 (12.6)	37 (78.7)	10 (21.3)	.52	
	Oxygen support (*no* *, *yes*)				109 (31.4)	84 (77.1)	25 (22.9)	.43	
	Duration of oxygen support, (days)			*<5* *	58 (54.2)	50 (86.2)	8 (13.8)	**.02**	**1**
				*≥5*	49 (45.8)	33 (67.3)	16 (32.7)		**3 (1.1‐7.8)**
	Death (*no* *, *yes*)				23 (6.18)	17 (73.9)	6 (26.1)	>0.99	

*Note*: HAdV positive and negative detection rates were established as a fraction of the total number of cases defined by each group. Saturation of O_2_ defined in the pediatric group, and confirmed when it is inferior to 94%.

Abbreviations: ARIs, acute respiratory infections; Body T°, body temperature; CRP, C‐reactive protein; ICU, Intensive Care Unit; HAdV, human *Adenovirus*; NRDS, neonatal respiratory distress syndrome.

a *P*‐value was calculated using the Pearson's *χ* ^2^ test or the Fisher's exact test on SPSS, where appropriate. A value of *P* ≤ .05 was considered as significant and is bolded in the Table.

b The odds‐ratio (OR) and 95% confidence interval [95% CI] were calculated using the parameter risk in crosstabs for variable with two categories or the binary regression model when the test exceeded two tails. OR and 95% CI with a value of *P* ≤ .05 (statistical significance) are bolded in the Table.

c The diagnosis of prematurity in neonates was estimated according to the amenorrhea week (confirmed if ≤37 amenorrhea week).

* Defines the reference group used in the statistical calculations.

### 3.2 Detection of HAdV and association with clinical and severity parameters

Respiratory pathogens were found in 526/583 of specimens (90.2%), from which 28.5% were caused by a single pathogen and 71.5% by at least two pathogens. HAdV was found in 114 patients (19.6%) and was the fourth most prevalent respiratory pathogen detected after HRV (52.1%), HRSV A/B (33.3%), and *S. pneumoniae* (31.0%). Most cases positive for HAdV were diagnosed in February 2014 (see Figure, Supplemental Digital Content 2 describing the seasonal distribution of HAdV infections in neonates, infants, and children hospitalized for ARIs in the Sousse area between October 2013 and December 2014 [n = 583]).

HAdV infection was more frequently observed in the pediatric than neonatal population (*P* < .001, OR = 3). Within neonates, adenoviral infections dominated in those aged more than 10 days (12.3% of the total HAdV positive samples, *P* = .04). Regarding the pediatric population, HAdV infection was fluctuating between age groups (*P* = .01): it was increasing in the groups 13 to 60 months (36.2%) and 4 to 6 months (32.8%) while decreasing in groups 7 to 12 months (25.0%) and 45 days to 3 months (17.6%). In 33.8% of pediatric cases with HAdV infection, a low saturation of oxygen (<94%) was recorded with no statistical association. However, a statistically significant association was found between HAdV infection and long duration of oxygen support (≥5 days), (32.7%, *P* = .02). Although hospitalized due to ARIs, HAdV infected neonates had, in addition, digestive symptoms (*P* = .02), and HAdV infection in pediatric patients was found to be statistically associated with gastroenteritis (*P* < .01) (Table 2).

### 3.3 Predictive adenoviral loads and impact on clinical and severity parameters

The association of clinical data and severity of ARIs with adenoviral loads (mean and median HAdV‐Ct‐values) showed no statistical significance, except for anemia, which increased significantly with higher adenoviral loads (see Table, Supplemental Digital Content 3 illustrating the comparison between HAdV‐Ct values from positive multiplex qRT‐PCR based on Ct mean = 33.4 and median = 34.6 and establishes the statistical associations between HAdV‐Ct values, clinical data, and severity of ARIs).

### 3.4 HAdV coinfections and association with severity of ARI

Among 114 HAdV infections, seven single infections were found (6.1%). In 107 cases, coinfection with at least one other pathogen was detected (93.9%), among which HRV (50.9%), *S. pneumoniae* (34.2%), HRSV A/B (34.2%), and the HCoV group (including HCoV‐229E, HCoV‐OC43, HCoV‐HKU1, and HCoV‐NL63, 21.9%) dominated. Coinfections with HMPV A/B (15.8%), EV (14.0%), HPeV (11.4%), and HBoV (10.5%) were less frequent. Coinfections with other viruses were rarely detected.

The statistical associations between HAdV coinfections and severity of infection were evaluated for HRV, HRSV A/B, *S. pneumoniae*, and HCoV group and are given in Table 3. HAdV‐*S. pneumoniae* was the only combination found to be associated with death (five fatal cases with HAdV‐*S. pneumoniae* positive coinfection vs two fatal cases positive for HAdV (one monoinfection and one case together with another virus, but negative for *S.pneumoniae)*. Due to the small sample size, no statistical calculations were performed.

**Table 3:** Statistical associations between human *Adenovirus* coinfections with other respiratory infectious agents and predictive severity factors

Patients data		HAdV‐HRV negative	HAdV‐HRV positive	*P* *	HAdV‐HRSV A/B negative	HAdV‐HRSV A/B positive	*P* *	HAdV‐*S. pneumoniae* negative	HAdV‐*S. pneumoniae* positive	*P* *	HAdV‐HCoVs negative	HAdV‐HCoVs positive	*P* *
Total (/114)		**56**	**58**	…	**80**	**34**	…	**75**	**39**	…	**89**	**25**	…
Admission	Neo	16 (28.6)	5 (8.6)	**<.01**	14 (17.5)	7 (20.6)	.69	16 (21.3)	5 (12.8)	.26	18 (20.2)	3 (12.0)	.56
	Ped	**40 (71.4)**	**53 (91.4)**		66 (82.5)	27 (79.4)		59 (78.7)	34 (87.2)		71 (79.8)	22 (88.0)	
Prematurity		**6 (15.0)**	**13 (24.5)**	.26	14 (21.2)	5 (18.5)	.77	15 (25.4)	4 (11.8)	.11	14 (19.7)	5 (22.7)	.76
Admission to ICU		**4 (7.1)**	**6 (10.3)**	.74	**5 (6.3)**	**5 (14.7)**	.16	6 (8.0)	4 (10.3)	.73	7 (7.9)	3 (12.0)	.45
Ventilation		**3 (5.4)**	**6 (10.3)**	.49	6 (7.5)	3 (8.8)	>.99	3 (4.0)	6 (15.4)	.06	6 (6.7)	3 (12.0)	.40
Death**		**3 (5.4)**	**4 (6.9)**	…	**3 (3.8)**	**4 (11.8)**	…	**2 (2.7)**	**5 (12.8)**	…	5 (5.6)	2 (8.0)	…

*Note*: The percentages were calculated as a fraction of the total number of HAdV coinfections defined by each category (negative and positive coinfections). Data increasing with positive HAdV coinfections were represented in the Table in bold.

Abbreviations: HAdV, human *Adenovirus*; HCoVs, human *Coronavirus* group including HCoV‐229E, HCoV‐HKU1, HCoV‐OC43, and HCoV‐NL63; HRV, human *Rhinovirus*; HRSV A/B, human *Respiratory Syncytial virus A/B*; ICU, Intensive Care Unit; Neo, neonatology ward; Ped, pediatric ward; *S. pneumoniae, Streptococcus pneumoniae*.

* *P*‐value was calculated using the *χ* ^2^ test or Fisher's exact test on SPSS, where appropriate. A value of *P* ≤ .05 was considered as significant and is bolded in the Table.

** No statistical associations were evaluated for death due to the small number of identified cases (solely seven cases).

### 3.5 Type identification of HAdVs and phylogenetic analysis

**Figure 1:**
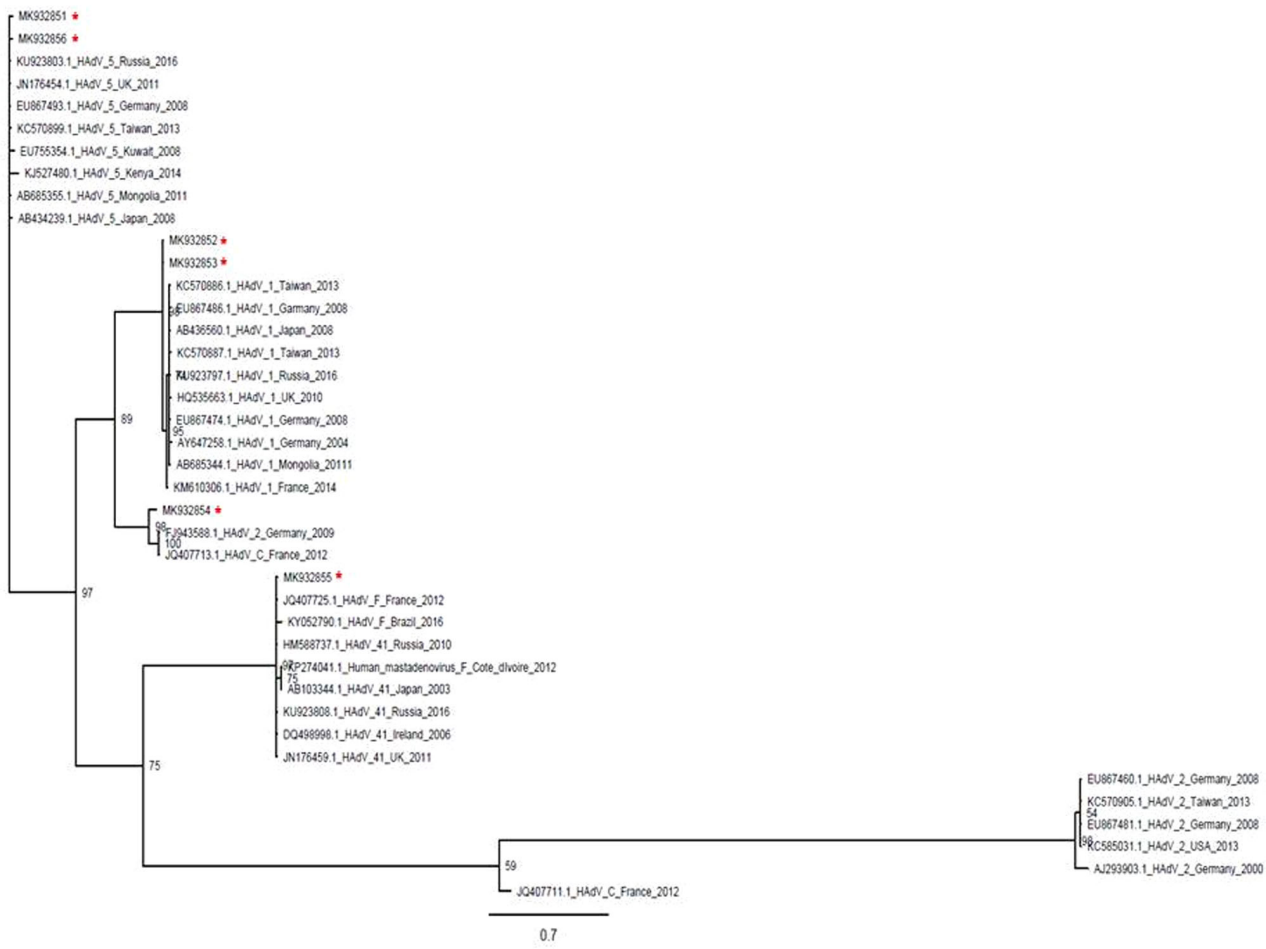
Phylogenetic analysis of the partial hexon gene from human *Adenovirus*‐positive specimens obtained between October 2013 and December 2014 in the area of Sousse, Tunisia. The nucleotide reference sequences of the archived partial hexon gene of HAdV‐C and ‐F species and respectively types 2, 1, 5, and 41 were obtained from GenBank and clustered according to the degree of homology (≥98%; Mr Bayes v3.2.2). Strains identified in this study were deposited on GenBank under accession numbers MK932851‐MK932856 and are accordingly labeled in this figure. HAdV, human *Adenovirus*; HAdV‐C, human *Adenovirus* species C; HAdV‐F, human *Adenovirus* species F

Table 4 summarizes the identified specimens. Among the HAdV positive samples, 12 were successfully typed and sequenced (mean adenoviral Ct‐values = 24.6, median adenoviral Ct‐values = 25.6) and revealed a predominance of HAdV‐C species and two specimens identified as HAdV‐B3 species. The severity of infection in these identified species consists of saturation of oxygen less than 94%, hospitalization for more than or equal to 7 days, bacterial superinfection, two cases admitted in the ICU and infected by HAdV‐C2, and a single fatality identified as HAdV‐C5 monoinfection. The two control specimens (Innsbruck) were identified as HAdV‐F41 (Supplemental Digital Content 1). Six from these sequences were deposited on GenBank under the accession numbers MK932851 to MK932856 and included in the phylogenetic tree. All sequences were variable suggesting the circulation of different HAdV strains, which shared more similarities to strains circulating in Europe or Asia than those from Africa (Figure 1).

**Table 4:** Clinical profile and predictive severity factors of human *Adenovirus* identified types from patients hospitalized for acute respiratory infections between 2013 and 2014

Patient ID	Age (mo)^a^	Underlying disease	Clinical diagnosis^b^	Severity and complication of ARIs^c^	Coinfections	Ct‐value^d^	Typing^e^	Sequencing/AN
***1***	5	Asthma	Bronchiolitis	Hospitalization for 13 d, saturation of O_2_ < 94%	HRV and *S. pneumoniae*	23.56	…	HAdV‐C type 1/MK932852
***2***	57	Asthma, passive smoking	Bronchiolitis	Hospitalization for 10 d, saturation of O_2_ < 94%	HBoV	26.17	…	HAdV‐C type 1/MK932853
***3***	23	…	Bronchiolitis	Admission to ICU (3 d), dehydration	HRV and HBoV	14.18	HAdV‐2	HAdV‐C type 2
***4***	23	…	Bronchiolitis	Admission to ICU (2 d), apnea	HRV, HMPVA/B, and HBoV	27.12	HAdV‐2	HAdV‐C type 2/MK932854
***5***	19	Prematurity	Bronchiolitis and laryngitis	Bacterial superinfection	*S. pneumoniae*	26.94	…	HAdV‐C type 5/MK932851
***6***	1	…	Bronchiolitis and rhinitis	Hospitalization for 16 d, bacterial superinfection	…	23.71	…	HAdV‐C type 5/MK932856
***7***	6	…	Bronchiolitis and rhinitis	Bacterial superinfection	HRV	25.02	HAdV‐3	HAdV‐B type 3
***8***	3	Asthma	Bronchiolitis	Hospitalization for 9 d, oxygen support (5 d), dehydration, bacterial superinfection	HRSV A/B, *S. pneumoniae*, and PeV	24.46	…	HAdV‐C type 1
***9***	4	Passive smoking	Bronchiolitis	…	HMPVA/B and EV	27.29	HAdV‐2	HAdV‐C type 2
***10***	6	Passive smoking	Bronchiolitis	Hospitalization for 10 d, nosocomial infection, **death** f	HRSV A/B and *S. pneumoniae*	22.81	…	HAdV‐C type 5
***11***	8	Asthma, passive smoking	Bronchiolitis and rhinitis	bacterial superinfection	HRV	26.60	HAdV‐3	HAdV‐B type 3
***12***	7	Passive smoking	Bronchiolitis, pharyngitis, and gastroenteritis	Hospitalization for 15 d, nosocomial infection	HCoV‐229E	27.76	HAdV‐2	HAdV‐C type 2

*Note*: The saturation of O_2_ was defined in the pediatric group and was confirmed when it is inferior to 94%.

Abbreviations: AN, Accession Number; CRP, C‐reactive protein; EV, *Enterovirus* genus; F, female; HAdV, human *Adenovirus*; HBoV; human *Bocavirus*; HCoV‐229E, human *Coronavirus* 229E; HMPV A/B, human *Metapneumovirus* A/B; PeV, *Parechovirus* genus; HRV, human *Rhinovirus*; ICU; Intensive Care Unit; M, male, *S. pneumoniae, Streptococcus pneumoniae*.

a The patient's age was described in month(s).

b Clinical diagnosis of patients and infections (laryngitis, rhinitis, pharyngitis, and gastroenteritis) associated with bronchiolitis.

c All the HAdV identified types presented symptoms of severe ARIs including mostly long duration of hospitalization, bacterial superinfection, admission to ICU, oxygen support, saturation of O_2_ < 94%, and death.

d The adenoviral Ct‐values were established from the multiplex qRT‐PCR.

e As primers used for HAdV typing by conventional PCR were not able to characterize all HAdV types, only HAdV types 2 and 3 were identified. Sequencing reactions established other HAdV including types 5 and 1.

f HAdV infection associated with fatality.

## 4 DISCUSSION

Earlier studies in Tunisia have reported HAdV infection rates of 19.5%^17^ and 25.8%^14^ in children with community‐acquired LARIs, and conform to this study which detected HAdV in 19.6% of tested samples. Thus, HAdV‐detection in the respiratory tract is important and should be considered in clinical settings. Indeed, throughout the world, HAdV has been shown to cause between 5% and 15% of UARIs and about 5% of LARIs in children.^6^, ^29^ This study has discovered that HAdV seems to increase the risk of low oxygen saturation and the duration of oxygen support in the pediatric environment which is in line with other reports showing that HAdV is associated with severe clinical manifestations.^30^, ^31^ In Africa, HAdV was associated with influenza‐like illnesses in several countries like Morocco, Egypt, Cameroon, and Senegal.^13^, ^32^, ^33^, ^34^ However, the severity of respiratory infections was variable. In Cameroon, HAdV was not found to be associated with severity.^33^ Conversely, in Senegal, HAdV infection was statistically correlated with myalgia, cough, diarrhea, headache, rhinitis, and pharyngitis.^34^ In the present study, a statistically significant association was found between HAdV infection and the manifestation of gastroenteritis and digestive symptoms. About twothird of infected cases needed oxygen support for more than 5 days; although, less than the half the cases negative for HAdV required long duration of oxygen support. This finding was supported by other reports indicating that children with severe HAdV infection including acute respiratory distress syndrome and respiratory failure need prolonged mechanical support in the form of either mechanical ventilation or extracorporeal life support and that early extracorporeal membrane oxygenation intervention for children should be considered.^35^ Another study reported that severe HAdV type 55 infection in adults causes rapid progression of respiratory failure with a high failure rate for noninvasive positive pressure ventilation and invasive mechanical ventilation. This failure rate may result to a large area of consolidation that induces a severe shunt in the lung, which will lead to a lack of response to positive pressure ventilation. For patients with severe acute respiratory distress syndrome, extracorporeal membrane oxygenation should be considered a better choice for oxygenation.^36^

Studies indicate that HAdV coinfections with other viral/bacterial agents may cause more severe disease. In this study, 93.9% of all HAdV specimens were coinfected with at least one other pathogen. The association between adenoviral coinfections with other viral agents and clinical data or severity of ARIs determined by this study, showed no association, suggesting that viral superinfection had no additional pathogenic role, supporting other findings.^37^ On the contrary to viral coinfections detected in this study, more severe infections were described in combination with HAdV‐*S. pneumoniae*, which seems to increase the risk of death. In corroboration to our finding, it was demonstrated that HAdV, particularly types 1, 2, 3, and 5 enhance pneumococcal adherence to the human respiratory epithelial cell culture due to an upregulation of receptors for *S. pneumoniae*.^38^ By this mechanism, HAdV‐associated ARIs may favor pneumococcal infections, which lead to severe diseases and deaths in patients under 5 years of age.

The role of HAdV in the pathogenesis of ARIs in still not clear. The high rate of coinfections found in this study and by others initiates the discussion as to whether HAdV contributes to the manifestation of ARIs. Indeed, some authors hypothesize that the multiplex molecular methods can simultaneously identify a wide range of pathogens, which are putative bystander infections/reactivations with no or limited causative role for the pathogenesis.^8^, ^39^ Though the total number of HAdV single infections in our study is too small (solely seven cases) to be statistically analyzed, HAdV infection is likely to be the causative agent for ARIs in these cases, as no other viral pathogen was detected. To provide a conclusive support for the hypothesis that HAdV is definitely implicated in ARIs, a larger patient cohort needs to be analyzed, and bacteria should be included.

Besides correlations with the severity of respiratory diseases, other approaches have been proposed for a better understanding of the role of HAdV in ARIs. The determination of viral loads and specific Ct‐values has been suggested to be considered.^40^ According to Esposito et al,^41^ low Ct‐values and thus high viral loads had a trend towards more complex disease in HAdV‐C respiratory infection. Moreover, a study done in Kuwait found that 75% of patients with HAdV severe respiratory infection had Ct‐values less than 30 cycles.^42^ In this study, no correlation could be established between the severity of ARI and Ct‐values except an increasing risk of anemia. This lack of association could be related to the insufficient number of specimens with high adenoviral loads. Some authors suggested performing a cell culture for specimens with high Ct‐values to increase the number of HAdV particles,^43^ however, the significance of low adenoviral loads in respiratory samples remains unclear and needs further and larger studies.

Within HAdV positive samples, only 12 were successfully typed and sequenced revealing one of the study limitations. Similarly, studies from Cameroon^29^ and China^44^ that sequenced specimens based on a partial hexon gene showed successful sequencing of under 11% of the samples. The difference in the sensitivity of qPCR used for HAdV‐detection could justify these results. Indeed, Kenmoe et al^33^ found a significantly low viral load of non‐typed samples with standard PCR compared to successfully typed samples (mean Ct‐values of 28.9 vs 15.4, respectively). In this study, the mean Ct‐values for non‐typed samples were 34.4 against 24.6 for successfully typed samples.

Most of the HAdV‐positive infections detected in this study were HAdV‐C followed by HAdV‐B. A report from Cameroon found that HAdV‐B infections were more common and severe than HAdV‐C.^33^ On the contrary, studies conducted in Egypt and Senegal were consistent with our findings showing a predominance of HAdV‐C.^10^, ^34^ The most frequently identified types in this study were HAdV‐C1, ‐C2, and ‐C5. This data is in line with the epidemiology of HAdV worldwide, which found these types in children with ARIs.^45^ The HAdV‐C infected patients identified in the present study had severe clinical manifestations with one fatal case infected by HadV‐C5. A study in the United States determining the risk factors for severe HAdV infection found that HAdV‐C5 increased the risk of severe disease.^46^ In addition, HAdV‐B species including types 3 and 7, and in some cases types 14, 21, and 55, and HAdV‐E species with the single known type 4, were also associated with severe clinical forms in the pediatric population requiring hospitalization.^36^, ^47^, ^48^ Sequences identified in the present study were variable suggesting the circulation of different HAdV strains. Unfortunately, comparison with previous HAdV strains identified in ARIs in Tunisia was not possible as no Tunisian HAdV sequences were published. Here, only sequences concerning enteric or environmental HAdV were available.^19^, ^20^, ^49^, ^50^

Although the multiplex qRT‐PCR can detect simultaneously a wide range of respiratory pathogens, low viral loads are probably not indicative of viral pathogenesis. The standard PCR used for typing and sequencing is based on the hexon gene, which allows more specific differentiation of adenoviral subtypes. However, it has the disadvantage of low sensitivity and requires samples with high viral loads. In addition, the fewer number of HAdV genotypes and sequences covered by the present study are considered among the study limitations. This number should be increased by referring to cell culture for samples with low viral loads or/and increasing the sampling size of the study population.

## 5 CONCLUSION

This study provides the first molecular and epidemiological description of HAdV‐ARIs circulating in Tunisia. Bacterial coinfections, especially with *S. pneumoniae*, seem to play a role. On the contrary, coinfections of respiratory viruses with HAdV do not seem to increase the severity of ARIs. Further studies with larger sampling are needed to clarify the role of HAdV loads or adenoviral Ct‐values and the role of HAdV genotypes in ARIs.

## CONFLICT OF INTERESTS

The authors declare that there are no conflict of interests.

## Supporting information

Supporting information

Supporting information

Supporting information

## Data Availability

The data that support the findings of this study are available from the corresponding author upon reasonable request.
