# Supplementary figures and images for: Human adenoviruses associated with respiratory illness in neonates, infants, and children in the Sousse area of Tunisia
Source: J Med Virol. 2020 Aug 13;92(12):3081–92. doi: 10.1002/jmv.26375 (PMC7689715; doi:10.1002/jmv.26375)

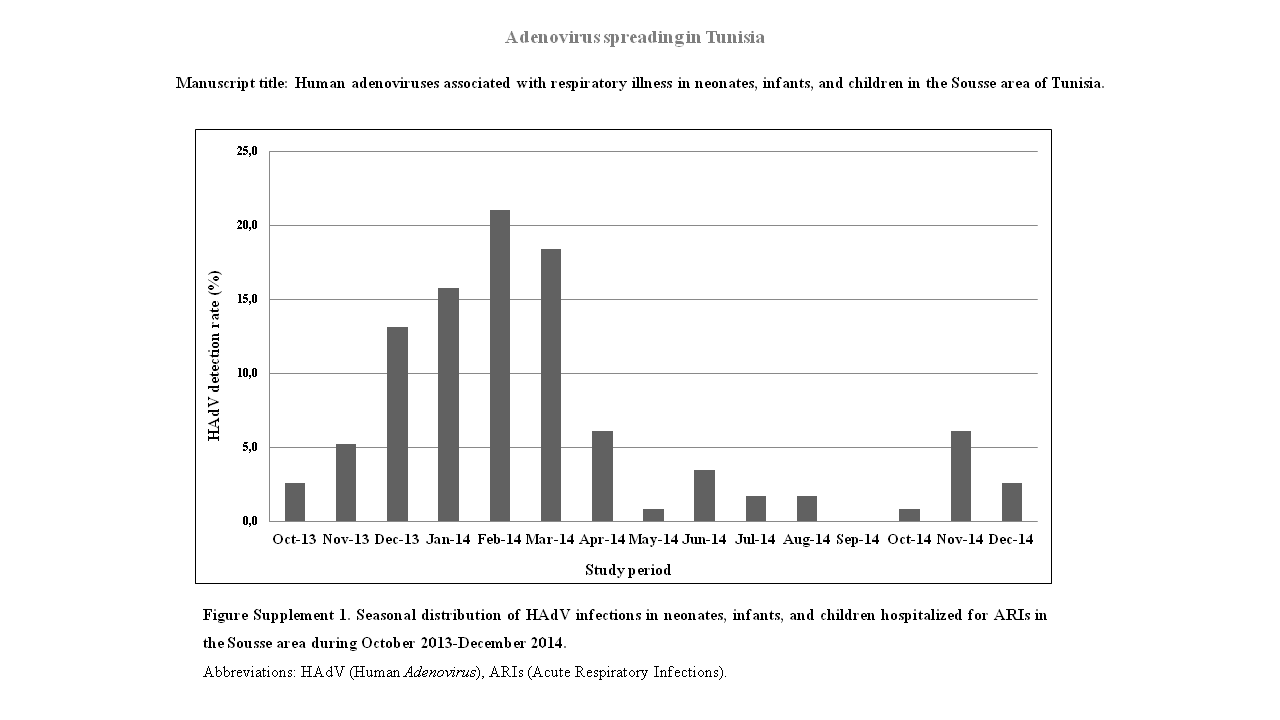

Supplement: Supplementary file 1 — Supporting information [file JMV-92-3081-s001.tif]
