# Supplementary material for: Human adenoviruses associated with respiratory illness in neonates, infants, and children in the Sousse area of Tunisia
Source: J Med Virol. 2020 Aug 13;92(12):3081–92. doi: 10.1002/jmv.26375 (PMC7689715; doi:10.1002/jmv.26375)
Supplement: Supplementary file 2 — Supporting information [file JMV-92-3081-s002.docx]

**Manuscript title: Human adenoviruses associated with respiratory illness in neonates, infants, and children in the Sousse area of Tunisia.**

**Table Supplement 1. List of HAdV reference sequences targeting a partial hexon gene and used in the construction of the phylogenetic tree (common length 632 bp).**

| **Accession No.** | **HAdV** | **HAdV type** | **Identification** | **Country/Year** | **Original length (bp)** | **Submitted/published** |
| --- | --- | --- | --- | --- | --- | --- |
| JQ407713 | **HAdV-C** | ***HAdV-C*** | HAdV-C isolate AdBx21 | France/2012 | 606 | Jan.2012/Berciaud et al., 2012 |
| JQ407711 |  |  | HAdV-C isolate AdBx75 | France/2012 | 611 | Jan.2012/Berciaud et al., 2012 |
| KC570886 |  | ***HAdV type 1*** | HAdV-C type 1 | Taiwan/2013 | 1519 | Feb.2013/ Wang et al., 2013 |
| EU867486 |  |  | HAdV-C type 1 | Germany/2008 | 1866 | Jul.2008/ Biere and Schweiger. 2010 |
| AB436560 |  |  | HAdV-C type 1 | Japan/2008 | 1418 | May.2008/Mizuta et al., 2009 |
| KU923797 |  |  | HAdV-C type 1 | Russia/2016 | 558 | Mar.2016/ Epifanova et al (unpublished) |
| HQ535663 |  |  | HAdV-C type 1 | UK/2010 | 546 | Oct.2010/ Al Qurashi et al., 2011 |
| KM610306 |  |  | HAdV-C type 1 | France/2014 | 1377 | Sep.2014/Cassir et al., 2014 |
| EU867474 |  |  | HAdV-C type 1 | Germany/2008 | 1482 | Jul.2008/ Biere and Schweiger. 2010 |
| AB685344 |  |  | HAdV-C type 1 | Mongolia/2011 | 1029 | Nov.2011/Tohma et al., 2012 |
| FJ943588 |  | ***HAdV type 2*** | HAdV-C type 2 | Germany/2009 | 1328 | Apr.2009/Biere and Schweiger. 2010 |
| EU867460 |  |  | HAdV-C type 2 | Germany/2008 | 1869 | Jul.2008/ Biere and Schweiger. 2010 |
| KC570905 |  |  | HAdV-C type 2 | Taiwan/2013 | 1546 | Feb.2013/Wang et al., 2013 |
| HM588735 |  |  | HAdV-C type 2 | Russia/2010 | 513 | Jun.2010/ Epifanova et al (unpublished) |
| KC585031 |  |  | HAdV-C type 2 | USA/2013 | 1525 | Feb.2013/Patel et al (unpublished) |
| AJ293903 |  |  | HAdV-C type 2 | Germany/2000 | 2910 | Sep.2000/Borcherding and Pring-Akerblom (unpublished) |
| KU923803 |  | ***HAdV type 5*** | HAdV-C type 5 | Russia/2016 | 558 | Mar.2016/ Epifanova et al (unpublished) |
| JN176454 |  |  | HAdV-C type 5 | UK/2011 | 546 | Jun.2011/Al Qurashi et al., 2011 |
| EU867493 |  |  | HAdV-C type 5 | Germany/2008 | 1830 | Jul.2008/ Biere and Schweiger. 2010 |
| KC570899 |  |  | HAdV-C type 5 | Taiwan/2013 | 1498 | Feb.2013/Wang et al., 2013 |
| EU755354 |  |  | HAdV-C type 5 | Kuwait/2008 | 617 | May.2008/Al-Rifaiy et al (unpublished) |
| KJ527480 |  |  | HAdV-C type 5 | Kenya/2014 | 522 | Mar.2014/Mbui et al (unpublished) |
| AB685355 |  |  | HAdV-C type 5 | Mongolia/2011 | 993 | Nov.2011/Tohma et al., 2012 |
| AB434239 |  |  | HAdV-C type 5 | Japan/2008 | 1097 | Apr.2008/Mizuta et al., 2009 |
|  |  |  |  |  |  |  |
| JQ407725 | **HAdV-F** | ***HAdV-F*** | HAdV-F isolate AdBx37 | France/2012 | 596 | Jan.2012/Berciaud et al., 2012 |
| KY052790 |  |  | HAdV-F isolate HMI/HP1/GO | Brazil/2016 | 611 | Oct.2016/da Paz and Souza (unpublished) |
| KP274041 |  |  | HAdV- F | Cote d’Ivoire/2012 | 2737 | Dec.2014/Pauly et al., 2015 |
|  |  | ***HAdV type 41*** | HAdV-41 reference sequence I* | Austria/2016 | 632 | -- |
| -- |  |  | HAdV-41 reference sequence II* | Austria/2016 | 632 | -- |
| HM588737 |  |  | HAdV-41 | Russia/2010 | 584 | Jun.2010/Epifanova et al (unpublished) |
| AB103344 |  |  | HAdV-41 | Japan/2003 | 1235 | Feb.2003/Li et al., 2004 |
| KU923808 |  |  | HAdV-41 | Russia/2016 | 552 | Mar.2016/ Epifanova et al (unpublished) |
| DQ498998 |  |  | HAdV-41 | Ireland/2006 | 541 | Apr.2006/Logan et al., 2006 |
| JN176459 |  |  | HAdV-41 | UK/2011 | 540 | Jun.2011/Al Qurashi et al., 2011 |

*Reference sequences identified in this study from Austrian children (HAdV-Austria_S1 and HAdV-Austria_S2) and used to optimize the AD1 and AD2 primers enrolled for sequencing reaction.

Abbreviations: Accession N° (Accession Number), HAdV (Human *Adenovirus*).
