# Supplementary material for: Human adenoviruses associated with respiratory illness in neonates, infants, and children in the Sousse area of Tunisia
Source: J Med Virol. 2020 Aug 13;92(12):3081–92. doi: 10.1002/jmv.26375 (PMC7689715; doi:10.1002/jmv.26375)
Supplement: Supplementary file 3 — Supporting information [file JMV-92-3081-s003.docx]

**Manuscript title: Human adenoviruses associated with respiratory illness in neonates, infants, and children in the Sousse area of Tunisia.**

**Table Supplement 2. Comparison between HAdV-Ct values (indicator of adenoviral loads) from positive multiplex qRT-PCR based on mean Ct-values (=33.4) and median Ct-values (=34.6) and statistical associations between HAdV-Ct values, clinical data, and severity of ARIs.**

|  | | **Mean Ct-values** | | ***P-value***** | **Median Ct-values** | | ***P-value***** |
| --- | --- | --- | --- | --- | --- | --- | --- |
|  | | **Ct ≤ 33.4** | **Ct > 33.4** |  | **Ct ≤ 34.6** | **Ct > 34.6** |  |
| **Total (/114 HAdV-positive cases)** | | **37 (32.5)** | **77 (67.5)** | **--** | **56 (49.1)** | **58 (50.9)** | **--** |
| **Clinical symptoms** | **Vomiting** *(no*, yes)* | 10 (27.0) | 16 (20.8) | .45 | 13 (23.2) | 13 (22.4) | .91 |
|  | **Diarrhea** *(no*, yes)* | 6 (16.2) | 12 (15.6) | .93 | 7 (12.5) | 11 (19.0) | .34 |
|  | **Anemia** *(no*, yes)^a^* | 21 (56.8) | 38 (49.4) | .46 | **35 (62.5)** | **24 (41.4)** | **.02** |
| **Predictive severity factors** | **Admission to ICU** *(no*, yes)* | 4 (10.8) | 6 (7.8) | .72 | 4 (7.1) | 6 (10.3) | .74 |
|  | **Oxygen support** *(no*, yes)* | 10 (27.0) | 26 (33.8) | .47 | 15 (26.8) | 21 (36.2) | .28 |
|  | **Ventilation** *(no*, yes)* | 2 (5.4) | 7 (9.1) | .71 | 3 (5.4) | 6 (10.3) | .49 |
|  | **Death** *(no*, yes)^b^* | **4 (10.8)** | **3 (3.9)** | .21 | **5 (8.9)** | **2 (3.4)** | .26 |

The percentages of clinical/severity data were established as a fraction of the total cases defined by each group (Mean Ct-values and Median Ct-values).

*Defines the reference group used in the statistical calculations.

**P-value was calculated using the Pearson’s chi-square (X^2^) test or the Fisher’s exact test on SPSS, where appropriate. A value of p≤ .05 was considered as significant.

^a^ The risk to develop anemia increases with the increasing adenoviral loads (low Ct-values, bolded in blue).

^b^ The majority of cases who ended fatal had the lowest Ct-values and are bolded in the Table. No statistical significance was found with death.

Abbreviations: HAdV (Human *Adenovirus*), ICU (Intensive Care Unit), Ct (cycle threshold).
